# Unsafe “crossover-use” of chloramphenicol in Uganda: importance of a One Health approach in antimicrobial resistance policy and regulatory action

**DOI:** 10.1038/s41429-021-00416-3

**Published:** 2021-03-19

**Authors:** Kayley D. McCubbin, John W. Ramatowski, Esther Buregyeya, Eleanor Hutchinson, Harparkash Kaur, Anthony K. Mbonye, Ana L. P. Mateus, Sian E. Clarke

**Affiliations:** 1 London School of Hygiene & Tropical Medicine, Keppel Street, Bloomsbury, London, WC1E 7HT United Kingdom; 2 The Royal Veterinary College, Hawkshead Lane, Hatfield, Hertfordshire, AL9 7TA United Kingdom; 3 Makerere University School of Public Health, New Mulago Hill Road, Kampala, Uganda

**Keywords:** Bacterial infection, Adverse effects

## Abstract

Since the introduction of antibiotics into mainstream health care, resistance to these drugs has become a widespread issue that continues to increase worldwide. Policy decisions to mitigate the development of antimicrobial resistance are hampered by the current lack of surveillance data on antibiotic product availability and use in low-income countries. This study collected data on the antibiotics stocked in human (42) and veterinary (21) drug shops in five sub-counties in Luwero district of Uganda. Focus group discussions with drug shop vendors were also employed to explore antibiotic use practices in the community. Focus group participants reported that farmers used human-intended antibiotics for their livestock, and community members obtain animal-intended antibiotics for their own personal human use. Specifically, chloramphenicol products licensed for human use were being administered to Ugandan poultry. Human consumption of chloramphenicol residues through local animal products represents a serious public health concern. By limiting the health sector scope of antimicrobial resistance research to either human or animal antibiotic use, results can falsely inform policy and intervention strategies. Therefore, a One Health approach is required to understand the wider impact of community antibiotic use and improve overall effectiveness of intervention policy and regulatory action.

## Description

Chloramphenicol is a phenicol class antibiotic with a narrow margin of safety used in small animal and human medicine [1]. Human oral bioavailability of crystalline chloramphenicol and chloramphenicol palmitate is approximately 80% [2], with rapid absorption and extensive distribution [3, 4]. Many countries have banned its use in livestock because human consumption of animal products containing chloramphenicol residues can cause idiosyncratic aplastic (non-regenerative) anemia [1, 5], which while uncommon, can be fatal due to its irreversible nature [1, 4–6]. Moreover, the adverse drug reaction is not dose-dependent and no safe consumption level has been established [4]. Additionally, chloramphenicol is considered to have potential carcinogenic effects [6]. Given these safety concerns, chloramphenicol is seldom used in humans and “reserved for severe, life-threatening infections for which other antibiotics are not available” [4].

In Uganda, National Clinical Guidelines reserve human oral chloramphenicol for use in hospitals while ear and ophthalmic preparations are permitted in primary healthcare facilities, including level II health centers [7, 8]. Enforcing antibiotic use (ABU) guidelines is difficult as Uganda’s formal healthcare sector is supplemented by private drug shops [9]. Drug shops provide crucial healthcare services in many low- and middle-income countries (LMICs) where drug shop vendors (DSVs) act as informal healthcare providers, advising patients on drug choice and dosing instructions [9–12]. Community-based drug shops often provide a more reliable drug stock than higher-level healthcare facilities, with convenient locations, lower costs,  and short waiting times [11]. Thus, drug shops represent an important source of antibiotics for many communities.

Although regulations prohibit Ugandan drug shops from selling antibiotics for human use without a prescription [13], this practice is widespread and seen in other LMICs [9–11, 14–16]. In contrast, veterinary drug shops can legally sell antibiotics over-the-counter which are used by farmers without veterinary consultation [17]. There is no formal chloramphenicol use ban in Ugandan livestock through The National Drug Policy and Authority Act, Section 64 (NDA, 2016), which regulates and restricts the use of classified drugs for agricultural purposes. However, the NDA does not permit veterinary drug shops to sell chloramphenicol for livestock use (Luwero district veterinary representative, personal communication). Nevertheless, our study provides evidence that livestock chloramphenicol use is common in Uganda and these activities should be investigated further.

A mixed qualitative and quantitative study conducted in Ugandan human and veterinary drug shops described human-intended chloramphenicol products being administered to livestock. This was discovered during a larger study investigating antibiotic availability and DSV views regarding treatment efficacy and antimicrobial resistance (AMR). All participation was voluntary and followed full informed consent and assurance of data confidentiality. Structured questionnaires including a complete antibiotic inventory were conducted with DSVs in 21 veterinary drug shops and 42 human drug shops, during May-June 2018. Three human DSVs declined participation due to concerns around reporting antibiotic sales. Six focus group discussions (FGDs) were held; two FGDs with veterinary DSVs and four with human DSVs.

No chloramphenicol was found in the antibiotic inventories of all 21 veterinary drug shops surveyed, indicating successful enforcement of restricting chloramphenicol sales through veterinary drug shops. Chloramphenicol formulations (oral, ear, and ophthalmic preparations) were found in 71.4% (30/42) of the human drug shops. While sector-specific antibiotic inventory lists are critical for understanding availability, these should not be considered strictly independent concerning ABU. It became evident during FGDs that off-label chloramphenicol use in livestock with human-licensed formulations was occurring. This was part of a wider phenomenon we term off-label “crossover-use”, in which human-intended antibiotic products were purchased from human drug shops for animal use, and similarly, veterinary antibiotic formulations were purchased for human use. Strikingly, descriptions of off-label crossover-use were frequently unprompted and depicted in all six FGDs as a widespread community practice. Use of chloramphenicol from human drug shops in poultry was described as exceedingly common, where “chloramphenicol is now know[n] as poultry capsules” (FGD III, Human DSVs), with commonly reported indications being fever and cough.

“…they will tell you that antibiotics meant for chicken don’t work and the human antibiotics tends to work on chicken more effectively” – FGD I, Human DSVs

Chloramphenicol crossover-use could be partially economically driven. Chloramphenicol sold in human drug shops provides a cheaper alternative to purchasing large packets of veterinary antibiotic formulations, in addition to more convenient sizes for small-scale farmers. In our inventory data, it cost 100 to 200 UGX (0.02 to 0.04 GBP^1^) per 250 mg chloramphenicol tablet. Comparatively, veterinary antibiotics were sold in 100 g packets and cost between 10,000 and 30,000 UGX (2.00 to 6.00 GBP), depending on the drug. If farmers are unaware of adverse health effects caused by chloramphenicol residue consumption, they may consider human-intended formulations as a viable and cost-effective alternative.

“One of the challenges is that one I have told you that veterinary drugs have no small packages and there are very many small scale farmers… our farmers are not commercial farmers, they are subsistence small scale farmers yet we do not have many of the vet drugs in such small packages.” – FGD II, Veterinary DSVs

Additionally, professional medical advice is not always accessible in sub-Saharan Africa, including Uganda, where veterinary services are fragmented [17]. Therefore, farmers are more likely to seek out alternative medical advice. Geographical access also represents a potential barrier to appropriate ABU. In our experience, human drug shops were much more abundant in rural regions; therefore, more accessible than their veterinary counterparts.

If chloramphenicol crossover-use is as rampant as FGDs described, the development of chloramphenicol resistance in poultry may have occurred. High levels of chloramphenicol-resistant *Escherichia coli* isolates were reported in Ugandan broiler chickens in 2010 (41% in Lira District, 42% in Kampala) [18]. Moreover, there is potential for cross-resistance development between chloramphenicol, florfenicol, linezolid, lefamulin, and tiamulin, important antibiotics in human and veterinary medicine, due to similar mechanism of action [19, 20]. However, the relationship between ABU and cross-resistance through alteration of similar binding sites is not straightforward [19], and cross-resistance between these antibiotics is rarely reported [20, 21]. However, florfenicol, linezolid, lefamulin, or tiamulin were not found in any human or veterinary drug shop surveyed nor were these reported to be used in livestock by DSVs. As chloramphenicol was not sold in any veterinary drug shops (0/21), but chloramphenicol was present in 71.4% (30/42) of human drug shops, it is conceivable that this resistance developed in part due to chloramphenicol crossover-use. Though, it also cannot be excluded that chloramphenicol or related resistance causing antibiotics were previously more accessible in veterinary drug shops. Nonetheless, mounting concern regarding the global threat of AMR underlines the urgency to reduce antibiotic misuse wherever possible. As resistance to antibiotics of public health importance can develop with continued chloramphenicol use, this highlights the need for monitoring and addressing antibiotic crossover-use in animals, as this is often overlooked.

Furthermore, chloramphenicol residues in animal products represent a danger to the community. Continued access to chloramphenicol for livestock in areas without rigorous antibiotic residue monitoring could lead to serious health challenges beyond AMR. High-levels of antibiotic residues (sulfamethazine and sulfadiazine) in eggs entering the food-chain in Uganda were detected [22], and concerns regarding antibiotic residues were raised in our FGDs. Veterinary DSVs claimed disregarding antibiotic withdrawal guidelines was “very common because we don’t have enforcement and regulatory mechanisms” (FGD II, Veterinary DSVs). This represents a potential consumer health risk that demands further investigation and attention from policymakers.

There is little information on continued chloramphenicol use in other countries. Only two studies were identified, both from Nigeria; Adebowale et al. [23], found that 44 of 103 (42.7%) of poultry farmers (layer hens) used chloramphenicol, though the chloramphenicol source was not stated. Omeiza et al. [24] confirmed that 15 out of 105 (14.5%) poultry farms reported using human-intended chloramphenicol products. It is clear that while the chloramphenicol ban in livestock is widespread, full adherence has not necessarily been achieved.

Reported off-label crossover-use highlights the importance of a One Health approach in AMR research and policy. If ABU research is limited to one sector or species, the full spectrum of community ABU would not be described. Likewise, if policy formulation, regulation, and enforcement aimed to address antibiotic misuse have a single sector focus, this could limit effectiveness.

Our findings suggest a lack of knowledge transfer regarding the potential adverse health effects of chloramphenicol residues to consumers and poor implementation of regulatory policy within and across sectors. It is unknown whether the rationale behind the sales ban was directly communicated to relevant stakeholders when initiated. Though from its continued widespread use, it appears this was not the case. Without fully informing the ultimate end users about the reason behind policy changes, bans may be ineffective.

These findings raise several policy considerations; including the need to communicate the rationale behind public health policy to end-users, concerns regarding food safety risks of chloramphenicol residues, and the importance of One Health approaches in research, and policy. These findings should be a call to action to alert farmers of the dangers of using chloramphenicol in livestock and an educational campaign should be initiated to communicate this urgent public health risk. DSVs have an important role in educating their customers to promote responsible ABU. As 50.0% and 23.8% of the human and veterinary DSVs, respectively, reported participating in continuing education workshops or courses, this represents an opportunity for discussing crossover-use. Furthermore, enforcement of the chloramphenicol ban should be extended to human drug shops to halt the continued use of chloramphenicol in livestock supported by awareness campaigns for the potential public health risks to enhance compliance. Safer treatment alternatives exist in the human health sector both within and external to the phenicol class of antibiotics [1, 3]. The need for improvement in education and enforcement was acknowledged by the DSVs.

“… I think it would be our role as both human and veterinary system to guide our clients before we sell the drugs to them, more so the human drugs would only be sold on prescription only but this open system of ours is the one causing problems where people come in as they wish, buy drugs and take.” – FGD II, Veterinary DSVs

Clearly, ABU is not limited to the intended species or sector, which poses a challenge for enforcement. Additionally, while chloramphenicol sales are banned specifically in veterinary drug shops, and along with all antibiotics in human drug shops, unrestricted chloramphenicol availability represents a gap in enforcement. Increasing knowledge regarding crossover-use implications may in part mitigate the issue; however, some enforcement will likely be required. Therefore, to be successful, enforcement capacity must be increased, both in regard to antibiotic sales and compliance to antibiotic withdrawal periods in livestock.

It is clear that the described findings represent a gap between policy intention and effective outcome, where a One Health approach is required. While inappropriate ABU contributes to AMR, without immediate action, this finding could lead to the continuation of additional adverse health consequences arising from antibiotic misuse.

## Footnotes

1 Conversion rate of 1.00UGX = 0.0002 GBP, December 2020
